# Dysregulated Iron Homeostasis in Atopic Dermatitis: Linking Iron Deficiency to Clinical Severity and Quality of Life

**DOI:** 10.3390/nu17233743

**Published:** 2025-11-28

**Authors:** Małgorzata Ponikowska, Alina Jankowska-Konsur, Łukasz Lewandowski

**Affiliations:** 1 Centre of General Dermatology and Oncodermatology, Wroclaw Medical University, 50-556 Wroclaw, Poland; alina.jankowska-konsur@umw.edu.pl; 2 Department of Biochemistry and Immunochemistry, Faculty of Medicine, Wroclaw Medical University, 50-368 Wroclaw, Poland; lukasz.lewandowski@umw.edu.pl

**Keywords:** iron deficiency, atopic dermatitis, inflammation

## Abstract

Background: Disturbed iron metabolism has been described in chronic diseases with pro-inflammatory/immune activation. This study aimed to characterize iron status in patients with atopic dermatitis (AD) and to examine its relationship with disease severity and quality of life. Methods: We prospectively enrolled 86 adult patients with moderate-to-severe AD. Clinical assessments included the Eczema Area and Severity Index (EASI), SCORing Atopic Dermatitis (SCORAD), and the Dermatology Life Quality Index (DLQI). Blood samples were collected for hematologic parameters and iron-related biomarkers, including serum iron, ferritin, transferrin, transferrin saturation (Tsat), soluble transferrin receptor (sTfR), and hepcidin. Associations between iron markers and clinical outcomes were evaluated using beta regression models with variable selection and stability analyses. Results: Abnormalities in circulating iron biomarkers indicating iron deficiency were prevalent in patients with AD: 45% of patients had low Tsat (<20%), 37% low ferritin, and 26% reduced serum iron, despite largely normal hemoglobin. Patients with pro-inflammatory activation (as evidenced by elevated high-sensitivity C-reactive protein (hsCRP) above 5 mg/L) displayed a pattern characterized by lower iron, Tsat and higher sTfR levels. In multivariable analyses, lower serum iron remained associated with worse DLQI scores, while higher transferrin was associated with greater disease severity (EASI, SCORAD). Conclusions: Iron deficiency without anemia was a common feature of moderate-to-severe AD and was associated with higher clinical burden. Dysregulated systemic iron homeostasis was associated with impaired quality of life and increased disease severity.

## 1. Introduction

Atopic dermatitis (AD) is a chronic inflammatory skin disease associated with complex immune dysregulation, systemic inflammation, and a substantial impact on quality of life. Traditionally regarded as a type 2 condition, AD is increasingly recognized as a systemic disorder extending beyond the skin, with mounting evidence linking it to metabolic disturbances, cardiovascular risk, and features of premature ageing [1,2,3].

Iron homeostasis plays a pivotal role not only in efficient erythropoiesis but also in cellular metabolism, including mitochondrial function, energetic processes, and enzymatic reactions essential for cell survival and immune competence [4,5]. Iron deficiency (ID) is the most prevalent micronutrient deficiency worldwide, affecting over two billion people, and remains the leading cause of anemia [6]. Importantly, ID may also occur without concomitant anemia, particularly in chronic inflammatory diseases, contributing to disease progression, cognitive impairment, and decreased quality of life, and has been associated with symptoms such as fatigue, reduced exercise tolerance, weakness, and impaired cognitive and metabolic function independent of hemoglobin levels [7].

In chronic inflammatory states, iron regulation becomes disrupted by pro-inflammatory cytokines, notably interleukin-6 (IL-6), which induces hepatic hepcidin production—the master regulator of systemic iron homeostasis [8,9,10]. Elevated hepcidin levels inhibit duodenal iron absorption and promote iron sequestration within the reticuloendothelial system, leading to restricted iron availability for cellular and immune functions [8,9]. This state, termed functional iron deficiency, may remain undetected when relying solely on standard indices such as hemoglobin or ferritin [9,10]. Consequently, comprehensive assessment using circulating indices—transferrin saturation (Tsat), soluble transferrin receptor (sTfR), and hepcidin levels—alongside markers of inflammation is required to identify subclinical ID in inflammatory settings [10].

Disturbances in iron homeostasis have been well documented in conditions such as heart failure [8], inflammatory bowel disease [11], diabetes mellitus [12], and more recently in dermatologic disorders with systemic inflammatory components, including psoriasis [13] and hidradenitis suppurativa [14]. However, little is known about iron metabolism in AD. Given the chronic inflammatory milieu characteristic of AD, subclinical ID may represent an under-recognized component of its systemic burden with potential implications for patient well-being and therapeutic strategies.

In this study, we aimed to characterize systemic iron homeostasis in patients with moderate-to-severe AD using circulating biomarkers and to explore their relationship with clinical severity and quality of life. We hypothesized that dysregulated iron metabolism in AD reflects a systemic manifestation of chronic inflammation and may contribute to disease burden and patient-reported outcomes.

## 2. Materials and Methods

### 2.1. Study Cohort

Patients with a diagnosis of atopic dermatitis (AD) were prospectively recruited at the Department of Dermatology, Venereology, and Allergology, Wroclaw Medical University, Poland.

All participants were adults (≥18 years old) with moderate-to-severe AD diagnosed according to established criteria (Hanifin and Rajka or UK Working Party) [15,16], with a disease duration of at least one year and clinically stable disease for at least four weeks prior to enrollment. All patients were biologic- and JAK-inhibitor–naïve. None had received systemic immunosuppressive therapy (including oral corticosteroids, cyclosporine, methotrexate, or azathioprine) within at least six months prior to enrollment—i.e., beyond the expected pharmacodynamic washout period for these agents. Topical corticosteroids and calcineurin inhibitors were permitted as maintenance therapy and recorded.

Exclusion criteria included: (i) any acute or chronic illness potentially affecting iron metabolism (e.g., known malignancy, active or chronic infection, severe chronic kidney disease, chronic cardiovascular disease, or hematologic disorders); and (ii) any treatment for anemia or iron deficiency within the preceding 12 months (oral or parenteral iron supplementation).

Disease severity was assessed using validated scoring tools (EASI, SCORAD), and quality of life was evaluated using the Dermatology Life Quality Index (DLQI) [17,18,19]. All assessments were performed during the same visit by trained dermatologists (EASI, SCORAD) or self-administered by patients (DLQI) before blood sampling. All blood samples were collected on the first day of hospital admission. At the time of venipuncture, no patient had received any systemic immunosuppressive therapy, systemic corticosteroids, cyclosporine, methotrexate, azathioprine, JAK inhibitors, or biologics. No systemic therapy was initiated prior to blood collection.

This sampling strategy ensured that all iron-related biomarkers were measured in a clinically relevant phase of active disease and before any systemic treatment could influence iron homeostasis or inflammatory markers. Specific allergen sensitization testing (allergen-specific IgE or skin prick tests) was not performed; therefore, patients were not phenotyped into IgE-associated (extrinsic) and non-IgE–associated (intrinsic) AD endotypes.

The study protocol was approved by the local ethics committee, and all participants provided written informed consent. The study was conducted in accordance with the principles of the Declaration of Helsinki [20].

### 2.2. Hematological Indices and Biomarkers of Iron Status

Venous blood samples were obtained in the morning after an overnight fast, following at least 15 min of supine rest.

Hematological parameters were measured using an automated analyzer (ADIVA 120; Siemens Healthcare Diagnostics, Deerfield, IL, USA). The parameters included hemoglobin concentration (g/dL), hematocrit (%), red blood cell count (×10^12^/L), mean corpuscular volume (fL), mean corpuscular hemoglobin (pg), and mean corpuscular hemoglobin concentration (g/L).

Anemia was defined according to the World Health Organization criteria as hemoglobin <12 g/dL in women and <13 g/dL in men [21].

Serum ferritin, iron, and total iron-binding capacity (TIBC) were measured using standard automated methods. Transferrin saturation (Tsat) was calculated as serum iron/TIBC × 100. Ferritin was determined by electrochemiluminescence immunoassay (Roche Diagnostics GmbH, Mannheim, Germany), and serum iron by colorimetric assay (Thermo Fisher Scientific, Waltham, MA, USA).

Iron deficiency (ID) was defined as serum ferritin <100 µg/L, or ferritin between 100–299 µg/L with transferrin saturation <20%, consistent with inflammation-adjusted thresholds recommended for chronic inflammatory diseases [8,21].

For extended characterization of iron status, serum hepcidin and soluble transferrin receptor (sTfR) were also measured. Hepcidin (ng/mL) was quantified using a commercial ELISA kit (Quantikine^®^, R&D Systems, Minneapolis, MN, USA), and sTfR (mg/L) was measured using immunonephelometry (Siemens Healthcare Diagnostics Inc., Deerfield, IL, USA).

Serum and plasma samples were aliquoted and stored at −70 °C until analysis.

Hepcidin is a peptide hormone produced in the liver and serves as the master regulator of systemic iron homeostasis [22]. It controls iron entry into the circulation by inhibiting intestinal absorption and macrophage iron release. When total body iron stores are depleted, hepatic hepcidin synthesis is suppressed, promoting iron absorption and mobilization from storage sites [23]. Conversely, pro-inflammatory cytokines such as interleukin (IL)-6 induce hepcidin overproduction, leading to reduced circulating iron and the development of anemia of inflammation [24].

Iron circulating in the blood is primarily bound to transferrin, which delivers it to tissues via interaction with transferrin receptor 1 (*TfR1*). Elevated soluble transferrin receptor (*sTfR*) levels indicate low intracellular iron and increased cellular demand, serving as a sensitive biomarker of functional iron deficiency [25,26].

### 2.3. Other Laboratory Assessments

Serum IL-6 was measured using commercially available Quantikine^®^ ELISA kits (R&D Systems, Minneapolis, MN, USA). Serum creatinine, aspartate aminotransferase (AST), alanine aminotransferase (ALT), and high-sensitivity C-reactive protein (*hsCRP*) were assessed by standard laboratory methods. Systemic pro-inflammatory activation was defined a priori as hsCRP > 5 mg/L, consistent with thresholds used to indicate clinically relevant low-grade inflammation and cardiometabolic risk in chronic inflammatory conditions [27].

### 2.4. Data Analysis Strategy

Statistical analysis was performed in R (version 4.3) using the tidyverse, glmnet, be-tareg, broom, car, and writexl packages [28,29]. Inference was based on a frequentist approach with α = 0.05. The clinical outcomes (DLQI, EASI, SCORAD) were rescaled to the open interval (0, 1) by dividing by their theoretical maxima (30, 72, and 103, respectively). None of the observations took exact values of 0 or 1, therefore standard beta regression models (logit link) were appropriate and zero/one-inflated variants were not required. Histograms of the rescaled outcomes showed unimodal distributions with mass away from 0 and 1, without marked floor or ceiling inflation, further supporting the use of standard beta regression. All analyses used a complete-case approach: rows with missing values in any variable entering a given model were excluded.

Continuous variables were centered around clinically relevant thresholds:

- Serum iron: 60 µg/dL
- Ferritin: 30 µg/L
- sTfR: 1.76 mg/L (upper limit of normal)
- Transferrin: 360 mg/dL (upper limit of normal)
- Tsat: 20%

All other continuous predictors (e.g., IgE, hsCRP, RBC, HGB, HCT) were centered at their sample medians to facilitate interpretation of intercepts and marginal predictions. IL-6 was analyzed as an additional inflammatory biomarker in descriptive summaries and in a Spearman correlation with hsCRP, but was not included as a predictor in the multivariable models.

Iron markers were compared between subgroups defined by hsCRP ≤ 5 mg/L vs. >5 mg/L using the Mann–Whitney U test.

Multivariate modeling included:

1. Penalized screening (LASSO):
  A five-fold cross-validated LASSO was applied to the logit-transformed outcomes (log(y/(1 − y))) to select candidate predictors. Penalized predictors were internally z-standardized; age and sex were included as unpenalized covariates (penalty factor = 0). Candidate predictors comprised age, sex, IgE, iron biomarkers (iron, ferritin, transferrin, sTfR, hepcidin), hematologic indices (RBC, HGB, HCT), and hsCRP.
2. Final model:
  Variables selected at step 1 were entered into a beta regression with logit link. We report coefficients β (logit scale), standard errors, Wald z statistics, *p*-values, and the precision parameter φ. Importantly, iron metabolism was modelled only as continuous values, rather than binary iron-deficiency categories, to preserve statistical information.
3. Stability assessment:
  Stability of variable selection was evaluated by 100-fold bootstrap resampling. In each bootstrap sample, CV-LASSO screening was repeated and selection frequencies (%) were computed. Additionally, for each final beta-regression model, non-parametric bootstrap distributions of regression coefficients were generated (500 resamples). For each predictor we report the median bootstrap coefficient and its percentile-based 95% CI (2.5th–97.5th). Multicollinearity among retained predictors was assessed using variance inflation factors (VIF).
4. Model-based marginal predictions:
  For clinical interpretability, we present model-based marginal predictions of DLQI, EASI, and SCORAD on their natural scales (0–30, 0–72, 0–103). Predictions were computed by:
  (a) estimating mean responses on the logit scale;
  (b) applying the inverse logit transformation;
  (c) back-transforming to the natural clinical range;
  (d) deriving 95% CIs using the delta method from the model’s variance–covariance matrix.

Given the sample size relative to the number of candidate predictors, we did not include interaction terms (e.g., hsCRP × iron biomarkers) in the models, as such specifications would be statistically underpowered and likely unstable. The primary aim of the modelling was inference rather than development of a standalone prediction tool; accordingly, we did not compute optimism-corrected global performance measures. Cross-validated penalization was used solely as a dimensionality-reduction step to stabilize multivariable estimates. Regression coefficients (β) and their standard errors are reported with three significant figures to ensure comparability across predictors measured on heterogeneous scales (e.g., IgE, serum iron, transferrin). Predicted values and marginal changes (Δ outcome) are shown using four decimal places (.4f), reflecting the 0–1 scale of beta-regression predictions. For predictors whose effects are extremely small, three significant figures were retained instead of .4f to avoid presenting rounded zeros. These conventions were applied consistently across all model tables.

## 3. Results

### 3.1. Baseline Characteristics of Studied Population

We enrolled 86 patients (46 [54%] men) with median age 29 years (interquartile range [IQR]: 23–39 years). Disease burden was high, as evidenced by DLQI, EASI, and SCORAD in the range typical for moderate-to-severe atopic dermatitis (Table 1). Total IgE was markedly elevated with median 3180.0 IU/mL (IQR: 235.0–11,037.5 IU/mL) and inflammatory activity was rather modest with median hsCRP: 2.35 mg/L (IQR: 1.0–6.7 mg/L) and median IL-6: 0.32 pg/mL (IQR: 0.20–0.53 pg/mL).

### 3.2. Haematological Indices and Iron Biomarkers

Routine hematologic indices were largely within reference limits (Table 2). Anemia was present in 13 (15%) patients.

Iron biomarkers are presented in Table 2.

Applying pre-defined, clinically relevant thresholds of blood-borne iron indices characterizing state of iron deficiency, we found low transferrin saturation (<20%) in 39 patients (45%), elevated transferrin (>360 mg/dL) in 11 patients (13%), elevated soluble transferrin receptor (>1.76 mg/L) in 4 patients (5%) and low ferritin (<30 µg/L) in 32 patients (37%).

Using inflammation-adjusted guideline criteria (ferritin <100 µg/L, or ferritin 100–299 µg/L with transferrin saturation <20%), iron deficiency was present in 67 of 86 patients (78%). while absolute iron deficiency (ferritin < 30 µg/L) was present in 32 (37%) patients of whom 6 (19%) had anemia.

Twenty-seven (31%) patients demonstrated pro-inflammatory activation (pre-defined as hsCRP > 5 mg/L) and their iron biomarkers are presented in Table 3. Patients with pro-inflammatory activation had significantly lower serum iron (*p* = 0.005) and transferrin saturation (*p* = 0.017), alongside with higher sTfR (*p* = 0.026), with no significant differences in ferritin, hepcidin, or transferrin.

### 3.3. Modelling the Clinical Severity Scores

#### 3.3.1. Quality of Life Impairment (DLQI)

**Figure 1:**
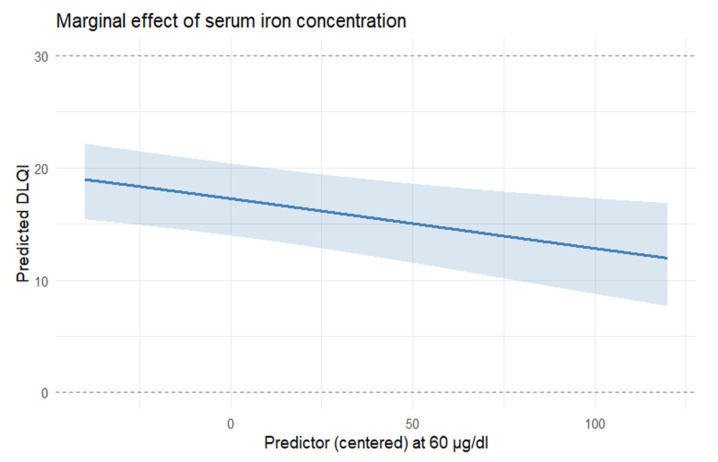
Association between iron concentration (in µg/dL) and DLQI—keeping the other features in the model stable. Model-based marginal predictions derived from the final multivariable beta-regression model (adjusted for age, sex, and covariates retained after LASSO screening). Shaded areas represent 95% confidence intervals obtained from the model’s variance–covariance matrix. N = 85 complete cases.

The model (Table 4) included prespecified covariates (age and sex) and candidate predictors (IgE, serum iron [Fe], ferritin, transferrin, RBC, hemoglobin, hepcidin, and sTfR). Increase in IgE was associated with higher DLQI (β = 0.0000249, SE = 0.00000667, z = 3.73, *p* < 0.001), while an increase in Fe had a negative association with DLQI (β = −0.00596, SE = 0.00222, z = −2.68, *p* = 0.007, Figure 1). The remaining coefficients were not statistically significant. The precision parameter indicated satisfactory concentration around the mean (φ = 6.7, SE = 0.968, z = 6.92, *p* < 0.001). Selection frequencies based on bootstrap were 98% for IgE, 96% for Fe, 43% for ferritin, 67% for transferrin, 44% for RBC, 38% for hemoglobin, 79% for hepcidin, and 62% for sTfR. Age and sex were prespecified (forced) covariates and were not included in the stability tally.

#### 3.3.2. Eczema Area and Severity Index Percentage (EASI)

**Figure 2:**
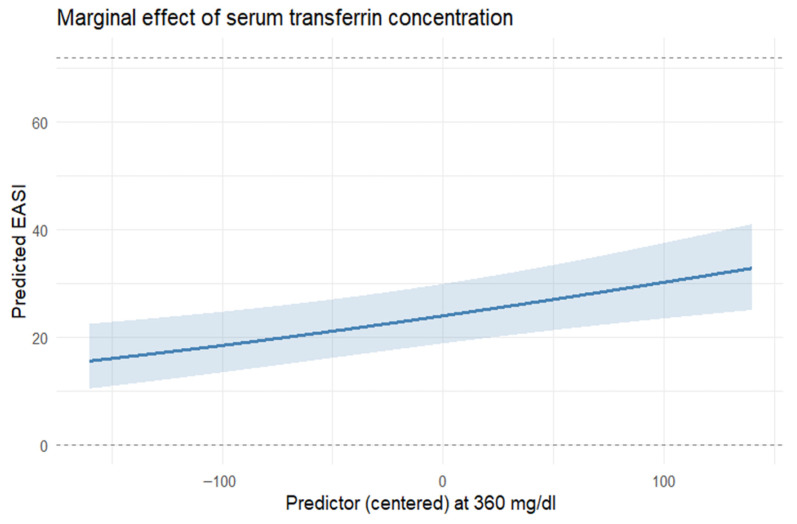
Association between transferrin concentration (in mg/dL) and EASI—keeping the other features in the model stable. Model-based marginal predictions derived from the final multivariable beta-regression model (adjusted for age, sex, and covariates retained after LASSO screening). Shaded areas represent 95% confidence intervals obtained from the model’s variance–covariance matrix. N = 85 complete cases.

The model (Table 5) included prespecified covariates (age and sex) and candidate predictors (IgE, serum iron [Fe], ferritin, transferrin, hematocrit [HCT], soluble transferrin receptor [sTfR], and hsCRP). Age was positively associated with EASI (β = 0.0134, SE = 0.00664, z = 2.02, *p* = 0.044). IgE was positively associated with EASI (β = 0.0000212, SE = 0.00000502, z = 4.23, *p* < 0.001). Transferrin showed a positive association (β = 0.00369, SE = 0.00109, z = 3.39, *p* = 0.001, Figure 2). HCT had a negative association (β = −0.0648, SE = 0.0222, z = −2.92, *p* = 0.004). sTfR also had a negative association (β = −0.829, SE = 0.238, z = −3.49, *p* < 0.001). The remaining coefficients were not statistically significant. The precision parameter indicated satisfactory concentration around the mean (φ = 10.7, SE = 1.58, z = 6.79, *p* < 0.001). Selection frequencies were 100% for IgE, 91% for transferrin, 88% for HCT, 87% for sTfR, 84% for Fe, 62% for hsCRP, and 56% for ferritin. Age and sex were prespecified (forced) covariates and were not tallied in the stability assessment.

#### 3.3.3. SCORing Atopic Dermatitis Percentage (SCORAD)

**Figure 3:**
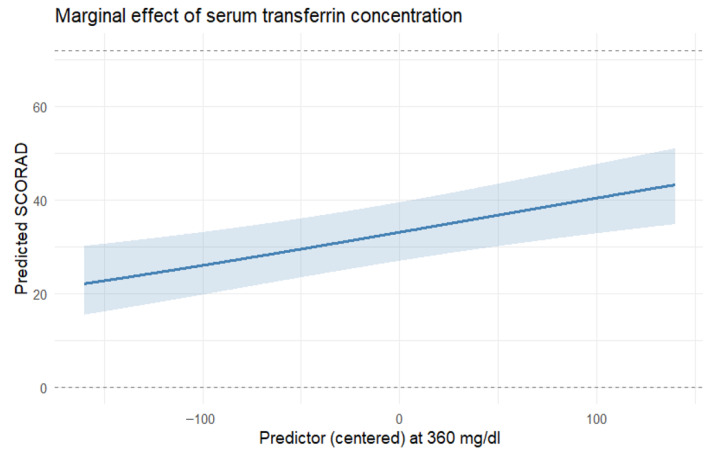
Association between transferrin concentration (in mg/dL) and SCORAD—keeping the other features in the model stable. Model-based marginal predictions derived from the final multivariable beta-regression model (adjusted for age, sex, and covariates retained after LASSO screening). Shaded areas represent 95% confidence intervals obtained from the model’s variance–covariance matrix. N = 85 complete cases.

The model (Table 6) comprised prespecified covariates (age and sex) and candidate predictors (IgE, serum iron [Fe], transferrin, RBC, hematocrit [HCT], soluble transferrin receptor [sTfR], and hsCRP). IgE was positively associated with SCORAD (β = 0.0000235, SE = 0.00000536, z = 4.37, *p* < 0.001). Transferrin showed a positive association (β = 0.00407, SE = 0.00111, z = 3.68, *p* = 0.0002, Figure 3). sTfR had a negative association (β = −0.817, SE = 0.240, z = −3.40, *p* <0.001). The remaining coefficients were not statistically significant. The precision parameter indicated satisfactory concentration around the mean (φ = 10.6, SE = 1.56, z = 6.8, *p* < 0.001). Selection frequencies were 100% for IgE, 88% for transferrin, 87% for sTfR, 85% for Fe, 70% for HCT, 64% for hsCRP, and 57% for RBC. Age and sex were prespecified (forced) covariates and were not included in the stability tally.

#### 3.3.4. Investigating Possible Multicollinearity Between the Main Effects in the Three Multivariate Beta-Regression Models

Variance Inflation Factors (VIFs) were computed for all predictors entering the final DLQI, EASI, and SCORAD models.

Iron-related biomarkers demonstrated uniformly low VIF values (Table 7), indicating that physiological correlations among ferritin, serum iron, transferrin, sTfR, and hepcidin did not meaningfully inflate variance estimates nor destabilize regression coefficients. Importantly, these VIF ranges fall well below commonly accepted thresholds for problematic multicollinearity.

Higher VIF values (~4–5) were observed only for erythropoietic variables (RBC, HGB, HCT), reflecting expected physiological collinearity within the red-cell axis. These variables served purely as adjustment covariates rather than primary determinants of atopic-dermatitis severity; consequently, their collinearity is clinically expected and methodologically acceptable.

Taken together with the bootstrap-derived selection stability (Table 4, Table 5 and Table 6), these findings confirm that the associations between iron biomarkers and clinical severity scores were not driven by multicollinearity.

#### 3.3.5. Sensitivity Analysis—Investigating the Uncertainty of Explored Multivariate Associations

Bootstrap resampling identified a small set of predictors with genuinely stable effects across models (Table 8). IgE showed a consistently positive and stable association with all three outcomes (DLQI, EASI, SCORAD). Among iron-related biomarkers, transferrin demonstrated a stable positive effect in the EASI and SCORAD models, while sTfR showed a stably negative association in the EASI and SCORAD models, while in the DLQI model its bootstrap interval narrowly crossed zero (borderline stability). In addition, hepcidin exhibited a stable negative effect in the DLQI model, and HCT showed a stable negative effect in the EASI model.

All remaining predictors—including serum iron, ferritin, RBC, HGB, hsCRP, and HCT in the other outcomes—displayed borderline or clearly unstable bootstrap intervals, with confidence limits crossing zero. These effects should therefore be interpreted as imprecise and not reproducibly detected by the model given the sample size and collinearity structure.

## 4. Discussion

In this study, we provide a systematic characterization of systemic iron homeostasis in patients with moderate-to-severe AD. Our results indicate that this condition is associated with marked abnormalities in circulating iron biomarkers indicative of iron deficiency (ID). Low transferrin saturation, reduced serum iron, and decreased ferritin were prevalent in AD, despite hemoglobin and standard hematinic indices largely remaining within the reference range. Nearly half of the cohort met criteria for low transferrin saturation, 37% had reduced ferritin, and 26% had low serum iron. Importantly, alterations in iron status were significantly associated with both clinical severity (EASI, SCORAD) and patient-reported quality of life (DLQI), suggesting the potential clinical relevance of dysregulated iron metabolism in AD.

The pattern of altered iron metabolism observed in our cohort parallels findings reported across a spectrum of chronic inflammatory disorders, where impaired iron handling has emerged as a hallmark of systemic inflammation. Similar mechanisms have been described in heart failure [30], inflammatory bowel disease [31], and diabetes mellitus [32], as well as in dermatologic diseases with systemic inflammatory components such as psoriasis [13] and hidradenitis suppurativa [14]. In psoriasis, low iron availability has been correlated with disease severity and systemic inflammatory activity, while in hidradenitis suppurativa, impaired iron metabolism has been linked to chronic inflammation and metabolic comorbidities. To date, however, iron status in AD has not been systematically evaluated, despite accumulating evidence that AD extends beyond the skin and is associated with systemic immune activation, metabolic dysfunction, and features of premature vascular ageing [33,34]. Our study therefore fills an important knowledge gap and suggests that iron deficiency may represent an underrecognized component of the AD phenotype.

The mechanistic link between chronic inflammation and iron dysregulation is well established. Proinflammatory cytokines, particularly interleukin-6 (IL-6), stimulate hepatic production of hepcidin, the master regulator of iron homeostasis [35]. Hepcidin inhibits ferroportin-mediated iron export from enterocytes and macrophages, leading to decreased dietary absorption and sequestration of iron in the reticuloendothelial system. This cascade results in functional iron deficiency, characterized by limited iron availability for cellular processes despite apparently normal or even elevated total body stores.

In our study, patients displayed only modest systemic inflammation, with IL-6 levels within the normal range and hsCRP elevated (>5 mg/L) in 31% of cases. Notably, patients with higher inflammatory activation had lower serum iron and transferrin saturation and higher sTfR, consistent with early functional iron deficiency, though ferritin and hepcidin were not significantly different between groups.

The potential clinical relevance of abnormal iron homeostasis indicative of systemic iron deficiency in AD is suggested by its associations with both disease severity and quality of life. We found that lower serum iron remained associated with greater DLQI impairment after adjustment for other covariates, while higher transferrin levels correlated with more severe eczema scores (EASI and SCORAD). Iron is essential for mitochondrial bioenergetics, enzymatic redox reactions, and immune regulation [36,37,38]. Deficiency can impair skin barrier integrity, enhance oxidative stress, and exacerbate fatigue and pruritus. These findings are consistent with the concept that systemic metabolic disturbances may contribute to the overall disease burden in AD. Whether correcting subclinical ID in AD could improve patient-reported outcomes or influence disease mechanisms remains an intriguing hypothesis warranting prospective interventional studies. A prospective, randomized study would be necessary to determine whether targeted correction of iron deficiency improves clinical outcomes in AD. Such a trial should enroll biologic-naïve adults with moderate-to-severe AD and laboratory evidence of systemic iron deficiency, randomizing participants to iron supplementation versus standard care. Primary functional endpoints may comprise validated clinical indices (EASI, DLQI) over 12–24 weeks, with safety monitoring for oxidative stress, infection risk, and eczema flares. This conceptual framework can parallel large randomized trials in chronic inflammatory and cardiometabolic diseases, where intravenous ferric carboxymaltose safely corrected iron deficiency and improved patient outcomes [39]. Whether similar inflammation-adjusted iron repletion protocols can be adapted to dermatologic settings with proinflammatory background to test symptomatic benefits needs to be evaluated.

Several limitations should be acknowledged. This was a single-center study with a moderate sample size, which may limit generalizability. The absence of a healthy control group precludes direct comparison with normal iron homeostasis. Furthermore, the cross-sectional design prevents causal inference about the relationships between iron status and AD severity or quality of life. In addition, residual confounding by unmeasured nutritional, metabolic, or hormonal factors cannot be entirely excluded. Lastly, we did not assess the impact of systemic or biologic therapies on iron metabolism, which may be relevant for future research. An additional methodological limitation is that we did not formally test interactions between inflammatory activity (hsCRP or IL-6) and iron biomarkers. Although biologically plausible, such models were statistically infeasible in this dataset: each beta-regression model already included 7–8 continuous predictors plus prespecified covariates, resulting in 9–10 parameters for only 86 complete cases. Introducing interaction terms would have substantially inflated model dimensionality and produced non-identifiable or unstable estimates—consistent with the coefficient variability already observed in our 500-iteration bootstrap. For this reason, our inference focuses on main-effect associations, and we avoid over-interpreting potential effect modification by inflammatory status.

Importantly, the bootstrap analysis demonstrated that several key associations were consistently stable across models, including the positive effect of IgE (all models) and the inverse association of sTfR (EASI and SCORAD), as well as a stable negative association between serum iron and DLQI. Other iron-related predictors showed only borderline or unstable behavior, indicating that further validation in larger cohorts is necessary.

## 5. Conclusions

In conclusion, our study identifies dysregulated iron homeostasis—indicative of iron deficiency—as a prevalent systemic feature of atopic dermatitis. These disturbances are linked to disease severity and impaired quality of life, suggesting that altered iron metabolism may represent a potentially modifiable systemic correlate of the AD phenotype. Future studies should explore whether targeted correction of iron deficiency can improve patient outcomes; our current observational data cannot address treatment effects. The observed associations between iron deficiency and disease burden suggest that iron status may be an overlooked comorbidity worth further investigation in AD.

## Figures and Tables

**Table 1:** Baseline characteristics of the study population.

Variable	N	Descriptive Statistics
Sex: male	86	46 (53.5%)
Age [years]	86	29.00 (23.00–39.00)
DLQI	86	18.00 (11.30–22.00)
EASI	86	30.40 (19.60–42.20)
SCORAD	86	55.60 (39.30–71.80)
IgE [IU/mL]	86	3180.00 (235.00–11,037.50)
hsCRP [mg/L]	86	2.35 (1.00–6.68)
IL-6 [pg/mL]	86	0.32 (0.20–0.53)

Median (IQR) and count (%) for quantitative and categorical variables, respectively; EASI—Eczema Area and Severity Index; SCORAD—SCORing Atopic Dermatitis; hs-CRP—high sensitivity C-reactive protein; IL-6—interleukin 6.

**Table 2:** Hematological and iron-related biomarkers in studied patients with atopic dermatitis.

Variable	N	Descriptive Statistics
Hb (g/dL)	86	14.00 (13.00–15.10)
HCT [%]	85	42.20 (39.20–44.70)
MCH [pg]	85	29.90 (28.60–30.70)
MCHC [g/dL]	85	33.20 (32.60–34.00)
MCV [fL]	85	89.50 (86.10–92.60)
RBC [×10^12^/L]	86	4.68 (4.32–4.99)
Fe [μg/dL]	86	84.00 (60.50–112.50)
Ferritin [μg/L]	86	40.80 (17.20–90.90)
sTfR [mg/L]	86	1.09 (0.90–1.26)
TIBC [μmol/L]	78	53.25 (46.53–60.40)
Transferrin [mg/dL]	86	270.00 (230.00–306.00)
Transferrin saturation [%]	86	21.00 (15.00–30.40)
UIBC [μmol/L]	64	34.50 (30.00–45.10)
Hepcidin [ng/mL]	86	8.93 (4.14–14.52)

Median (IQR) and count (%) for quantitative and categorical variables, respectively.

**Table 3:** Comparison of iron biomarkers in patients with vs. without proinflammatory stimulation (stratification by hsCRP, with a 5 mg/L cut-off).

Variable	hsCRP ≤ 5 mg/L (*n* = 59) Median (IQR)	hsCRP > 5 mg/L (*n* = 27) Median (IQR)	*p*
Fe [μg/dL]	93.30 (62.80–122.00)	60.9 (46.40–86.80)	0.005
Ferritin [μg/L]	42.70 (19.90–91.40)	35.00 (17.20–75.00)	0.413
Hepcidin [ng/mL]	9.56 (4.19–14.15)	7.09 (3.38–16.85)	0.696
sTfR [mg/L]	1.02 (0.89–1.23)	1.18 (1.06–1.31)	0.026
Transferrin [mg/dL]	271.00 (234.00–304.50)	264.0 (230.00–309.50)	0.798
Transferrin saturation [%]	23.70 (16.20–35.60)	16.60 (13.40–22.90)	0.017

**Table 4:** Beta regression model for DLQI.

Feature	βi	βi SE	Z	*p*	DLQI	Change in DLQI	Bootstrap Stability [%]
(Intercept)	0.3050	0.227	1.35	0.179	17.267	-	-
Age [years]	−0.00246	0.00815	−0.302	0.763	-	-	Forced covariate
Sex: male	−0.074	0.228	−0.325	0.745	-	-	Forced covariate
IgE [IU/mL]	0.0000249	0.00000667	3.73	<0.001	17.267	0.000182	98
Fe [μg/dL]	−0.00596	0.00222	−2.6800	0.007	17.223	−0.0437	96
Ferritin [μg/L]	0.00292	0.00228	1.28	0.201	-	-	43
Transferrin [mg/dL]	0.00224	0.0014	1.6	0.110	-	-	67
RBC [G/L]	0.292	0.322	0.908	0.364	-	-	44
HGB [g/dL]	−0.16	0.126	−1.27	0.204	-	-	38
Hepcidin [ng/mL]	−0.0283	0.0147	−1.93	0.053	-	-	79
sTfR [mg/L]	−0.536	0.313	−1.71	0.087	-	-	62
Φ	6.7	0.968	6.92	<0.001	-	-	-

**Table 5:** Beta regression model for EASI.

Feature	βi	βi SE	Z	*p*	EASI	Change in EASI	Bootstrap Stability [%]
(Intercept)	−0.688	0.177	−3.89	**<0.001**	24.0792	-	-
Age [years]	0.0134	0.00664	2.02	**0.044**	24.2944	0.2150	forced covariate
Sex: male	0.0181	0.184	0.099	0.921	-	-	forced covariate
IgE [IU/mL]	0.0000212	0.00000502	4.23	**<0.001**	24.0796	0.000340	100
Fe [μg/dL]	−0.00208	0.00178	−1.17	0.243	-	-	84
Ferritin [μg/L]	−0.000782	0.00137	−0.57	0.568	-	-	56
Transferrin [mg/dL]	0.00369	0.00109	3.39	**0.001**	24.1384	0.0591	91
HCT [%]	−0.0648	0.0222	−2.92	**0.004**	23.0521	−1.0300	88
sTfR [mg/L]	−0.829	0.238	−3.49	**<0.001**	12.9509	−11.1000	87
hsCRP [mg/L]	0.00371	0.00475	0.782	0.434	24.1387	0.0596	62
Φ	10.7	1.58	6.79	**<0.001**	-	-	-

**Table 6:** Beta regression model for SCORAD.

Feature	βi	βi SE	z	*p*	SCORAD	Change in SCORAD	Bootstrap Stability [%]
(Intercept)	−0.156	0.180	−0.866	0.386	47.4911	-	-
Age [years]	0.00192	0.00615	0.312	0.755	47.5403	0.0491	(forced covariate)
Sex: male	−0.00357	0.167	−0.021	0.983	47.3998	−0.0914	(forced covariate)
IgE [IU/mL]	0.0000235	0.00000536	4.37	**<0.001**	47.4917	0.000600	100
Fe [μg/dL]	−0.00233	0.00176	−1.32	0.186	47.4315	−0.0597	85
Transferrin [mg/dL]	0.00407	0.00111	3.68	**<0.001**	47.5953	0.1040	88
RBC [G/L]	−0.0332	0.261	−0.127	0.899	46.6426	−0.8480	57
HCT [%]	−0.038	0.0337	−1.13	0.261	46.5201	−0.9700	70
sTfR [mg/L]	−0.817	0.240	−3.4	**0.001**	28.2511	−19.2000	87
hsCRP [mg/L]	0.00433	0.00468	0.925	0.355	47.6020	0.1110	64
Φ	10.6	1.56	6.8	**<0.001**	-	-	-

**Table 7:** Variance Inflation Factors (VIF) for iron-related biomarkers in the three beta-regression models.

Iron Biomarker (Centered)	DLQI Model	EASI Model	SCORAD Model
Serum iron	1.49	1.42	1.42
Ferritin	3.43	-	1.85
Transferrin	1.52	1.44	1.41
sTfR	1.87	1.65	1.60
Hepcidin	2.84	-	-

**Table 8:** Sensitivity analysis with the use of Bootstrap.

DLQI model			
Predictor	β-hat	Bootstrap (median; 95% CI)	Stability
Ferritin	0.00292	0.00290 (−0.00236 to 0.00829)	borderline
Transferrin	0.00224	0.00231 (−0.000510 to 0.00529)	borderline
sTfR	−0.536	−0.565 (−1.23 to 0.00140)	borderline
IgE	0.0000248	0.000026 (0.0000140–0.0000390)	stable
Serum iron	−0.00595	−0.00588 (−0.0111 to −0.000720)	stable
Hepcidin	−0.0283	−0.0287 (−0.0543 to −0.00246)	stable
RBC	0.292	0.300 (−0.320 to 1.11)	unstable
HGB	−0.160	−0.182 (−0.487 to 0.0820)	unstable
SCORAD model			
Predictor	β-hat	Bootstrap (median; 95% CI)	Stability
Serum iron	−0.00233	−0.00213 (−0.00567 to 0.000830)	borderline
HCT	−0.0379	−0.0444 (−0.111 to 0.0210)	borderline
IgE	0.0000235	0.0000240 (0.0000160–0.0000370)	stable
Transferrin	0.00407	0.00395 (0.00102–0.00664)	stable
sTfR	−0.817	−0.798 (−1.32 to −0.107)	stable
RBC	−0.0332	0.000510 (−0.497 to 0.510)	unstable
hsCRP	0.00433	0.00421 (−0.0235 to 0.0196)	unstable
EASI model			
Predictor	β-hat	Bootstrap (median; 95% CI)	Stability
Serum iron	−0.00208	−0.00199 (−0.00524 to 0.00120)	borderline
IgE	0.0000212	0.0000216 (0.0000140–0.0000330)	stable
Transferrin	0.00369	0.00360 (0.00109–0.00604)	stable
HCT	−0.0648	−0.0649 (−0.124 to −0.00800)	stable
sTfR	−0.829	−0.815 (−1.34 to −0.159)	stable
Ferritin	−0.000781	−0.000719 (−0.00377 to 0.00181)	unstable
hsCRP	0.00371	0.00364 (−0.0227 to 0.0176)	unstable

β-hat denotes the coefficient from the beta-regression model (logit link). “Bootstrap (median; 95% CI)” refers to the median coefficient and its non-parametric 95% bootstrap confidence interval (2.5th–97.5th percentile) based on 500 resamples. “Stability” indicates the robustness of each predictor: stable = CI does not cross zero; borderline = CI touches or marginally crosses zero; unstable = CI widely includes zero. Age and sex were prespecified covariates and therefore omitted from the stability assessment.

## Data Availability

Data supporting the findings of this study are available from the corresponding author upon reasonable request. Access to the data is restricted due to ethical and legal considerations involving human participants.

## Abbreviations

The following abbreviations are used in this manuscript:

AD	Atopic dermatitis
CRP	C-reactive protein
DLQI	Dermatology Life Quality Index
EASI	Eczema Area and Severity Index
Fe	Serum iron
HCT	Hematocrit
hsCRP	High-sensitivity C-reactive protein
ID	Iron deficiency
IL-6	Interleukin-6
IQR	Interquartile range
SCORAD	SCORing Atopic Dermatitis
SD	Standard deviation
SE	Standard error
sTfR	Soluble transferrin receptor
TIBC	Total iron-binding capacity
Tsat	Transferrin saturation
